# Influence of halophytic hosts on their parasites—the case of *Plicosepalus acaciae*

**DOI:** 10.1093/aobpla/plu084

**Published:** 2014-12-16

**Authors:** Maik Veste, Henning Todt, Siegmar-W. Breckle

**Affiliations:** 1 Department of Ecology, University of Bielefeld, Universitätsstraße 25, D-33615 Bielefeld, Germany; 2 Institute of Botany, University of Hohenheim, Garbenstrasse 30, D-70599 Stuttgart, Germany

**Keywords:** Ion pattern, Loranthaceae, mistletoe, parasite, plasticity, salt stress, succulence.

## Abstract

Halophytes develop various morphological and physiological traits that enable them to grow successfully on saline substrates. Parasitic plants on halophytic hosts may also encounter salt stress. We investigated the mistletoe *Plicosepalus acaciae* (syn: *Loranthus acacia*; Loranthaceae), which occurs on 5 halophytic and at least 10 non-halophytic hosts in the Southern Arava Valley (Israel). *Plicosepalus acaciae* is a common parasite north of Eilat to the Dead Sea area and in the Jordan Valley. Morphological and physiological responses of *P. acaciae* to salinity were investigated by comparison of plants on halophytic with those on non-halophytic hosts. Ion patterns of different host–parasite associations were determined as was the development of leaf succulence at different growth stages. The leaf water content of *P. acaciae* increased and leaves developed succulence when growing on halophytic hosts, especially on *Tamarix* species, where leaf water content was three times higher than that on non-halophytic hosts and the leaf volume increased four to five times. The reason for increased succulence was a higher ion concentration of, and osmotic adjustment with, Na^+^ and Cl^−^. *Plicosepalus acaciae* showed a high morphological and ecophysiological plasticity, enabling it to cope with salt stress, and can be classified as a facultative eu-halophyte, which increases its halo-succulence according to the host. Host–parasite associations are a model system for the investigation of halophytes under different salt stress conditions.

## Abstract (precis)

Parasitic plants obtain nutrients, carbohydrates, ions and water from their host plants and develop special morphological features to survive on the living tissue of other plants. We investigated the mistletoe *Psilocephalus acaciae* on halophytic and non-halophytic hosts in the Arava Valley. When *P. acaciae* was parasitic on halophytic hosts, ions were transported by the transpiration stream in the parasite. Chloride and sodium induced the development of leaf succulence in the parasite. Increasing succulence is a morphological adaptation by the mistletoe to the increasing salt stress on halophytic hosts.

## Introduction

Halophytes develop various physiological and morphological adaptations to cope with high salt concentrations in soil and water (Waisel 1972; Breckle 2002; Flowers and Colmer 2008), and the regulation of uptake of Na^+^, K^+^ and Cl^−^ and transportation and storage of these ions in plant organs are key issues to understand life strategies (Breckle 1990). In combination with the physiological processes, morphological adaptations like leaf bladders, salt glands and succulence develop to mitigate the effects of high salt concentrations in the photosynthesizing tissue by desalinization of leaves or dilution or compartmentalization within leaf tissues (Breckle 1974, 2002; Schirmer and Breckle 1982; Freitas and Breckle 1992; Veste 2007*a*). Furthermore, the ion pattern and chemical composition of terrestrial halophytes are often characteristic of the species and family and only partly influenced by soil conditions (‘physiotype concept’; Albert 1982). For example, some desert halophytic members of the Aizoaceae are characterized by a selective hyper-accumulation of Na^+^ and Cl^−^ in their leaves even on soils with low salinity (Veste *et al*. 2004; Veste 2007*a*) as is also the case with the members of Amaranthaceae (Chenopodiaceae: see Veste and Breckle 2000; Albert 2005). On the other hand, members of the Poaceae are able to limit the uptake of Na^+^ (Albert 1982, 2005). This emphasizes the importance of active ion regulation and selective transportation of ions already in the root system.

An interesting question is what happens if the root system for such ion selection is missing, as in the case with parasitic plants? Is the pattern of ion accumulation between host and parasite mirrored to some extent? In this context, xylem-tapping mistletoes are a perfect model lacking a root system for selective ion uptake. Water and nutrient uptake is directly linked to their hosts (Glatzel and Geils 2009) through a haustorium, which connects the parasite with its host and allows the transportation of water, inorganic and organic compounds from the host’s transpiration stream directly into the parasite. While there has been considerable attention to nutrient exchange between the parasite and the host (e.g. Lamont 1983; Glatzel and Balasubramaniam 1987; Popp 1987), only little attention has been given to mistletoes growing on halophytic hosts and the influence of the salinity experienced by the host on the parasite. There are several reports from mistletoes on mangroves: *Phthirusa maritima* (Loranthaceae) on *Conocarpus erectus* and *Coccoloba uvifera* (Goldstein *et al.* 1989); *Lysiana subfalcata* on *Ceriops tagal* (Ullmann *et al.* 1985), *Loranthus rhamnifolius* on *Sonneratia alba* and *Loranthus sansibarensis* and *L. dregei* on *Lumnitzera racemosa* (Walter and Steiner 1936) and *Loranthus capitellatus* (Holtermann 1907). In these cases, the mistletoes on mangroves have a 0.2- to 3.5-fold higher Na^+^ concentration than their hosts (Lamont 1983; Goldstein *et al.* 1989). In South Africa and Namibia, of 19 mistletoes only *Tapinanthus oleifolius* (Loranthaceae) occurs on the halophytes *Tamarix usneoides* and *Salvadora persica* (Visser 1981; Popp *et al.* 1995), where it has a higher salt concentration in its xylem sap than the host. *Tapinanthus oleifolius* also increases its leaf succulence on the halophytic *T. usneoides* as well as on non-halophytic *Euphorbia virosa* (Veste 2007*b*), which is strongly correlated with the accumulation of inorganic ions (Popp *et al.* 1995). *Tamarix ramosissima* is an uncommon host for *Phoradendron californicum* (Loranthaceae) in North America, but the mistletoe is able to grow on the halophyte (Haigh 1996).

Another example for such a host–mistletoe association is *Plicosepalus acaciae* (syn: *Loranthus acaciae*; Loranthaceae) with an east-Sudanian distribution. The mistletoe occurs along the Arava Valley and some adjacent wadi systems north to the Jordan Valley and in some parts of Jordan (Shmida and Darom 1992; Qasem 2009, 2011). Within the Arava Valley, the highest density of *P. acaciae* can be found close to Yotvata (Todt *et al.* 2000), where most of the trees are parasitized by *P. acaciae*. The number of parasites decreases strongly a few kilometres north and south of Yotvata, and the movement pattern of the bird bulbul (*Pycnonotus xanthopygos*), as the main disperser of the red berries with sticky seeds, can explain the distribution of *P. acaciae* in the Arava Valley (Green *et al.* 2009). The most common hosts are Acacia trees because of their wide distribution (Munzbergova and Ward 2002; Bowie and Ward 2004). Other hosts for *P. acaciae* in the southern Arava Valley are trees and shrubs (*Atriplex*, *Nitraria* and *Tamarix*; Todt *et al.* 2000). On these hosts the parasite shows morphological and physiological plasticity as known for other halophytes (Breckle 2002) growing under different salt conditions. This high flexibility makes the different host–parasite associations of *P. acaciae* an interesting model system to understand adaptive plasticity to salt stress. We wanted to check how the parasite is able to cope with halophytic hosts growing under high salinity stress and the ecophysiological plasticity of ion accumulation that is involved. A special focus is given to ion uptake and its link with the development of leaf succulence at different growth stages on different hosts. We also compiled an updated checklist of the known host–parasite associations.

## Methods

**Figure 1.**
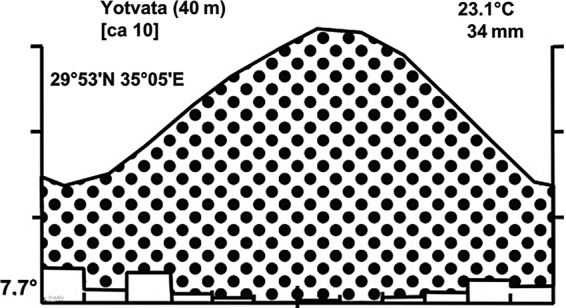
Climatic diagram of Yotvata (southern Arava, Israel) indicating the 12-months arid season.

Plant collections were made in May and in September 1997 in the southern Arava Valley at Yotvata and surroundings (29°53′N, 35°3′E, 40 km north of Eilat, Israel). The area is characterized by natural *Acacia*–*Tamarix* vegetation (Veste 2004) with partly mobile sand dunes, agricultural fields, date palm plantations and the nearby settlement Yotvata. Surrounding hills are hamada desert with scattered *Haloxylon salicornicum*. Average annual rainfall is 34 mm, which falls very irregularly, but predominantly in the winter season from November to March (Fig. 1). Herbarium material from the recorded mistletoe–hosts associations was kept in the Herbarium of the Department of Ecology, University of Bielefeld and in 2005 transferred to the Herbarium of Göttingen.

For investigations, leaves of 5–10 mistletoes were collected and, after checking fresh weight, were oven-dried (105 °C). Leaf area (determined with a Summasketch II digitizer tablet, Summagraphics Corp., Fairﬁeld, CT, USA) and leaf thickness (determined with a caliper) were measured from young, middle-aged and old leaves. Their number depended on availability and varied between 100 and 750. Old leaves were those showing initial signs of senescence (wilting or yellowing) on older branches (∼2 years old). Ash content was determined after heating at 600 °C in an oven. Succulence (*S*) was based on the organic dry matter (after Breckle 1976) and was calculated as follows:

$$S=g\;H_{2}O/g\;\mathrm{organic}\;d.m.=\mathrm{fw}-d.m./d.m.-\mathrm{ash}$$

Ion content was determined in hot water extracts with an atomic absorption spectrometer (AAS 2280, Perkin-Elmer, Waltham, MA, USA) with a C_2_H_2_ flame. Cl was determined with a micro-chlorocounter (Marius, Utrecht, NL). In all analytic determinations, there were three repetitions. If the relative standard deviation was >2 %, then additional checks were made.

Statistical analyses were performed with SPSS package 7.5.1. We checked the normality of distributions and variance of all samples with the Kolgomorov–Smirnow test (Köhler *et al.* 1996; Lozán and Kausch 1998). For correlation analysis we used Spearman's rank correlation coefficient (*r*’s). Significance of differences between samples fixed at the 0.05 probability level for all statistical tests was checked with the Mann–Whitney *U* test.

## Results

A list of the hosts of *P. acaciae* at the study site, Yotvata, in the Arava Valley and some other areas of the Middle East are given in Table 1. A high proportion of wild host species had reduced leaves and photosynthesis occurred mainly in stems. The hosts *Haloxylon persicum* and *Calligonum comosum* grew on sand dunes in Yotvata and other sandy areas in the Arava Valley. *Albizzia*, *Casuarina* and *Delonix* are introduced species and grew within the Yotvata settlement, whereas *Tamarix aphylla* is native to the northern Negev, but is planted in Yotvata as shelterbelts around fields and date palm plantations. The size of the mistletoe varied between the different hosts. The largest (and most) individuals of *P. acaciae* grew on *Acacia* and *Tamarix*, while only a few (and smaller) individuals occurred on *Atriplex* and *Haloxylon*. The highest number of *P. acaciae* in the area was on *Acacia tortilis*, *A. raddiana* and *Tamarix nilotica*, with no observable differences in density or preferences for non-halophytic or halophytic hosts.

**Table 1.** Checklist of non-halophytic and halophytic hosts of *P. acaciae* in the Arava Valley (Israel and Jordan). Hosts in Yotvata (Israel) are marked with YOT. W, species growing in natural habitas; Cv, cultivated species. Data source: (1) Post (1932), (2) Zohary (1966), (3) Täckholm (1974), (4) Feinbrun-Dothan *et al.* (1991), (5) Shmida and Darom (1992), (6) Veste and Breckle (1995); Todt *et al.* (2000), (7) Vaknin *et al.* (1996), (8) Kotschy (1861), (9) Qasem (2009, 2011).

Host species	Family		Reference/comments
Non-halophytic hosts			
*Acacia asak* (Forssk.) Willd.	Mimosaceae	Cv	9
*Acacia farnesiana* (L.) Willd.	Mimosaceae	Cv	9
*Acacia nilotica* (L.) Delile (=*A. arabica* (Lam. Willd.)	Mimosaceae	W	8, 9
*Acacia raddiana* Savi	Mimosaceae	W,YOT	1, 2, 3, 4, 5 (only *Acacia*), 7
*Acacia saligna* (Labill.) Wendl. (=*A. cyanophylla* L.)	Mimosaceae	Cv	9
*Acacia tortilis* (Forskk.) Hayne	Mimosaceae	W,YOT	1, 2, 3, 4, 5 (only *Acacia*), 6, 7
*Albizzia lebbeck Bentham*	Caesalpiniaceae	Cv	6
*Anagyris foetida* L*.*	Fabaceae	W	9
*Balanites aegyptiaca* (L.) Delile	Zygophyllaceae	Cv	4
*Calligonum comosum* L'Her.	Polygonaceae	W,YOT	6
*Capparis spinosa* L.	Capparidaceae	W	6, 9
*Casuarina cunninghamiana* Miq.	Casuarinaceae	Cv	6
*Casuarina equisetifolia* L.	Casuarinaceae	Cv	9
*Ceratonia siliqua* L.	Caesalpiniaceae	W	9
*Delonix regia* (Boyer ex. Hook) Rauf	Caesalpiniaceae	Cv	6
*Elaeagnus angustifolius* L.	Elaeagnaceae	W	8
*Ficus carica* L.	Moraceae	Cv	9
*Haloxylon persicum* Bunge	Chenopodiaceae	W,YOT	8
*Juglans regia* L.	Juglandaceae	Cv	9
*Melia azedarach* L.	Meliaceae	Cv	9
*Nerium oleander* L.	Apocynaceae	W	9
*Parkinsonia aculeata* L.	Caesalpiniaceae	Cv	9
*Pistacia atlantica* Desf.	Anacardiaceae	W	9
*Pistacia vera* L.	Anacardiaceae	Cv	9
*Poinciania gilliesii* Wall. ex Hook.	Caesalpiniaceae	Cv	9
*Prosopis chilensis* (Mol.)Stuntz.	Mimosaceae	Cv	9
*Prosopis farcta* Macbridge	Mimosaceae	W,Hazeva	Y. Vaknin pers. comm. in 6, 9
*Punica granatum* L.	Punicaceae	Cv	2
*Retama raetam* Webb & Berth	Fabaceae	W	9
*Rhamnus* spec. L.	Rhamnaceae	W	1
*Rhus tripartita* Grande	Anacardiaceae	W,YOT	4 (only *Rhus*), 9
*Ochradenus baccatus* Delile	Resedaceae	W	4, 5 (only *Ochradenus*), 6
*Pistacia atlantica* Desf.	Anacardiaceae	W	9
*Salix alba* L.	Salicaceae	W	9
*Ziziphus jujuba* Mill.	Rhamnaceae	Cv	9
*Ziziphus lotus* Lam.	Rhamnaceae	W,YOT	9
*Ziziphus spina-christi* (L.) Desf.	Rhamnaceae	W	1, 2, 3, 4, 5 (only *Ziziphus*), 7, 9
Halophytic hosts			
*Atriplex halimus* L.	Chenopodiaceae	W, YOT	2, 4 (only *Atriplex*)
*Nitraria retusa* Forsk.	Zygophyllaceae	W, YOT	4 (only *Nitraria*), 6
*Tamarix aphylla* (L.) Karsten	Tamaricaceae	Cv, W, YOT	2, 4, 5 (only *Tamarix*)
*Tamarix jordanis* Boiss*.*	Tamaricaceae	Cv, YOT	2, 4, 5 (only *Tamarix*)
*Tamarix nilotica* Bunge	Tamaricaceae	W, YOT	2, 4, 5 (only *Tamarix*), 6
*Tamarix pentandra* Pallas	Tamaricaceae	Cv	9

**Figure 2.**
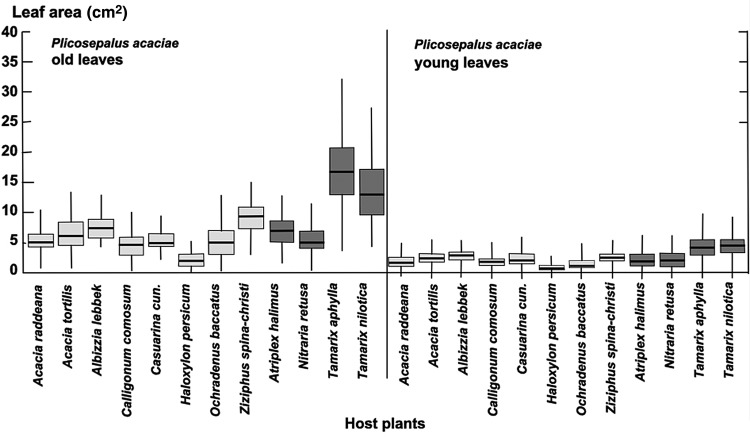
Leaf area (cm^2^) of young and old leaves of *P. acaciae* parasitic on non-halophytic (light grey) and halophytic (dark grey) hosts. Box plots indicate interquartile range, median (thick line) and total variation.

**Figure 3.**
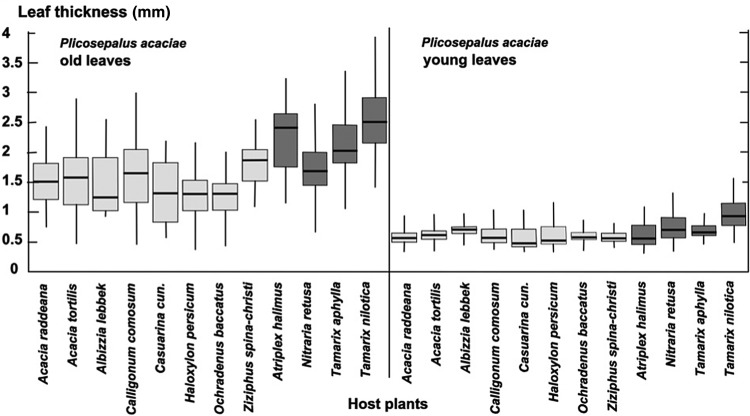
Leaf thickness (mm) of young and old leaves of *P. acaciae* parasitic on non-halophytic (light grey) and halophytic (dark grey) hosts. Box plots indicate interquartile range, median (thick line) and total variation.

**Figure 4.**
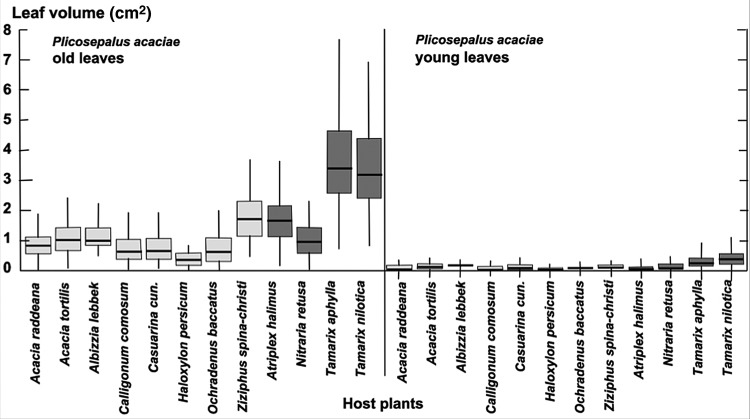
Volume (cm^3^) of young and old leaves of *P. acaciae* parasitic on non-halophytic (light grey) and halophytic (dark grey) hosts. Box plots indicate interquartile range, median (thick line) and total variation.

**Figure 5.**
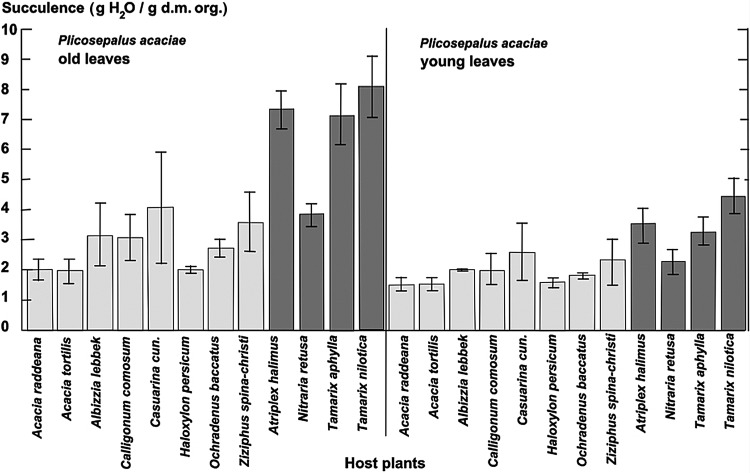
Succulence (g H_2_O g^−1^ d.m. org.) of young and old leaves of *P. acaciae* parasitic on non-halophytic (light grey) and halophytic (dark grey) hosts, with standard deviation.

We determined leaf area (Fig. 2), leaf thickness (Fig. 3) and thus leaf volume (Fig. 4) for *P. acaciae*, which varied according to host and increased considerably with leaf age. In old leaves, leaf volumes were up to 3-fold higher when parasitizing halophytic hosts in comparison with non-halophytic hosts. Leaf area, thickness and volume increased very significantly for *P. acaciae* on all *Tamarix* species (Figs 2–4) when compared with non-halophytic hosts. The maximum leaf area of *P. acaciae* on *T. nilotica* was 17.0 cm^2^ (median 13.0 cm^2^), whereas on *A. tortilis* the maximum leaf area was 8.1 cm^2^ (median 6.01 cm^2^). The leaf thickness of old leaves ranged from 1.2 to 1.85 mm (median 1.6 mm) on *A. tortilis* and from 2.15 to 2.80 mm (median 2.45 mm) on *T. nilotica*. An increase in the leaf thickness and succulence was also observed for old leaves of *P. acaciae* growing on the halophyte *Nitraria retusa* compared with the non-halophytic species, except that the differences were not significant on *Calligonum cumosum* and *Casuarina cunninghamiana*. Succulence, expressed as water content related to organic dry matter (Fig. 5), exhibited the same trends. The mean succulence of *P. acaciae* on *T. nilotica* was 7.99 ± 0.94 g H_2_O g^−1^ org. d.m., whereas on *A. tortilis* succulence was 1.94 ± 0.41 g H_2_O g^−1^ org. d.m. *Plicosepalus acaciae* developed succulent leaves also on *Atriplex halimus* (7.25 ± 0.56 g H_2_O g^−1^ org. d.m.) and *T. aphylla* (7.06 ± 0.88 g H_2_O g^−1^ org. d.m.).

**Figure 6.**
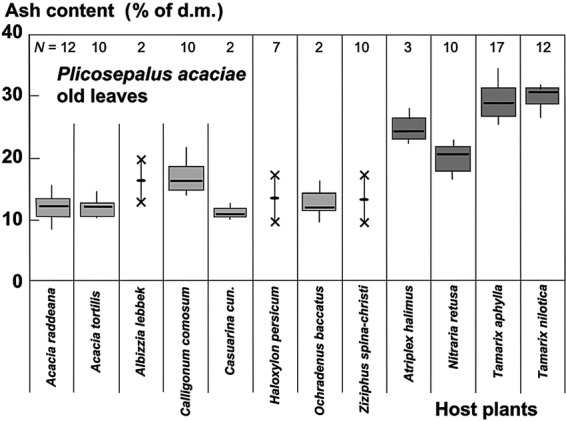
Ash content of old leaves of *P. acaciae* parasitic on non-halophytic (light grey) and halophytic (dark grey) hosts. Box plots indicate interquartile range, median (thick line) and total variation for *N* > 2.

**Figure 7.**
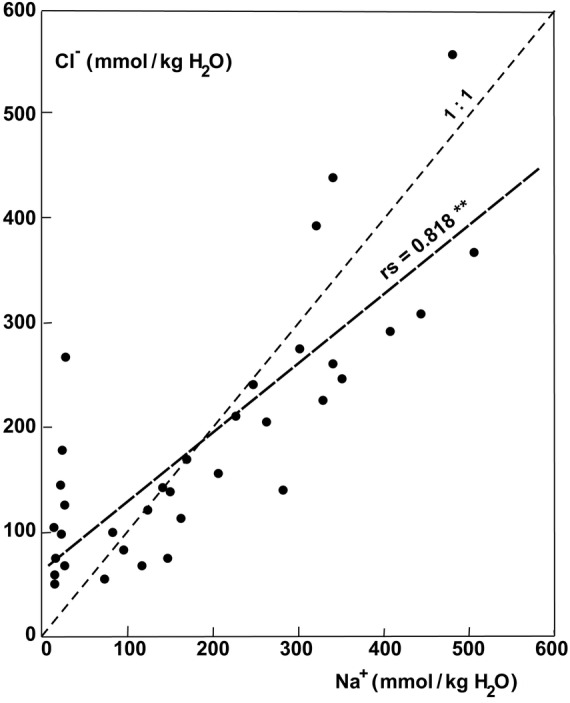
Correlation between Na^+^ and Cl^−^ concentration (mmol kg^−1^ H_2_O) in leaves of *P. acaciae* growing on non-halophytic and halophytic hosts.

**Figure 8.**
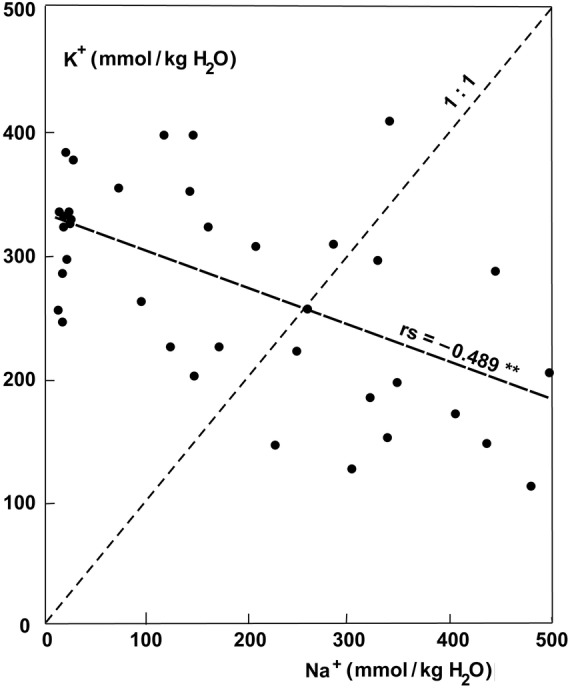
Correlation between Na^+^ and K^+^ concentration (mmol kg^−1^ H_2_O) in leaves of *P. acaciae* growing on non-halophytic and halophytic hosts.

**Figure 9.**
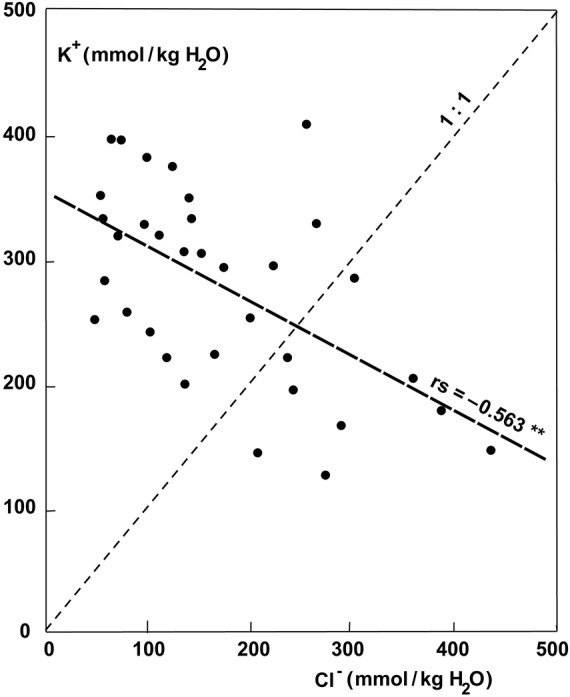
Correlation between K^+^ and Cl^−^ concentration (mmol kg^−1^ H_2_O) in leaves of *P. acaciae* growing on non-halophytic and halophytic hosts.

**Figure 10.**
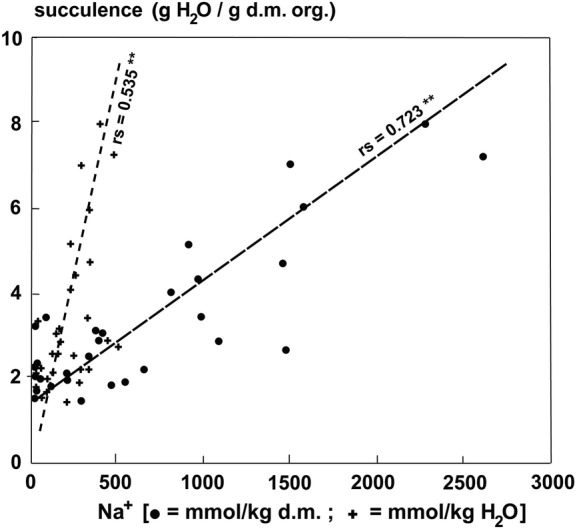
Correlation between succulence (g H_2_O g^−1^ d.m. org.) and Na^+^ concentration (dots: mmol kg^−1^ d.m.; crosses: mmol kg^−1^ H_2_O) in leaves of *P. acaciae* growing on non-halophytic and halophytic hosts.

**Figure 11.**
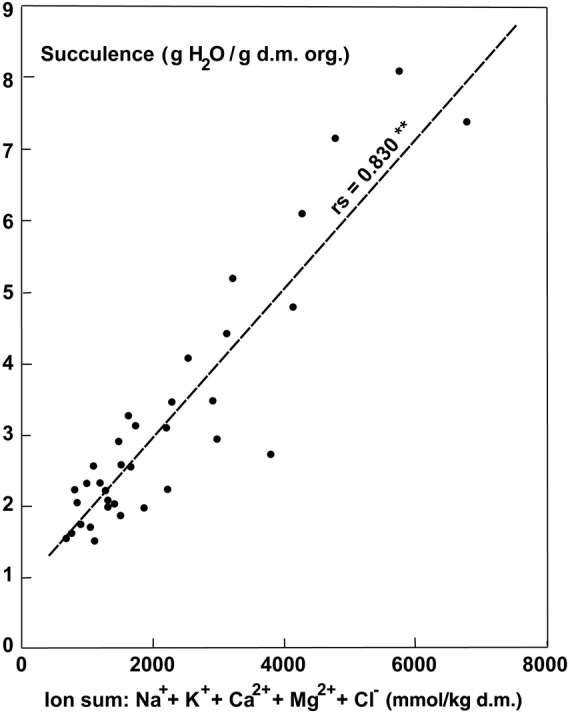
Correlation between succulence (g H_2_O g^−1^ d.m. org.) and the ion sum (Na^+^ + K^+^ + Ca^2+^ + Mg^2+^ + Cl^−^, mmol kg^−1^ d.m.) in leaves of *P. acaciae* growing on non-halophytic and halophytic hosts.

The ash content, which represents the sum of ions accumulated, exhibited much higher values in the parasites growing on halophytic than non-halophytic hosts (Fig. 6). The ash content of old leaves of plants growing on *T. nilotica* was 30.6 % compared with 12.1 % on *Acacia tortlis*. Accumulation of Na^+^ was similar to that of Cl^−^ at higher concentrations (Fig. 7). At lower concentrations, the uptake of Na^+^ could be very low and seemed to be better controlled than Cl^−^ uptake (Fig. 7). The uptake of Na^+^ and K^+^ was not strongly correlated but still definitely antagonistic (Fig. 8). Therefore, it follows that K^+^ uptake was also antagonistic to Cl^−^ (Fig. 9). Succulence of leaves of *P. acaciae* increased significantly with increasing leaf Na^+^ concentration (Fig. 10), and with an even stronger correlation if both Na^+^ and Cl^−^ were taken into account (Fig. 11).

**Figure 12.**
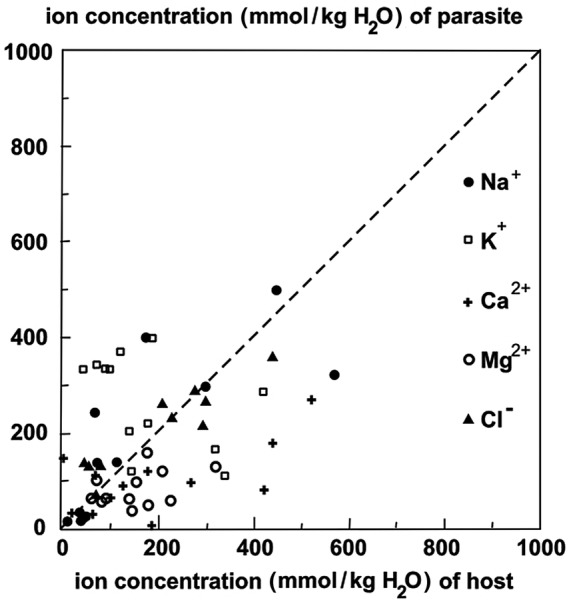
Ratio of ion concentration parasite/host for the five ions, calculated from the H_2_O values (mmol kg^−1^). The values for *A. halimus* are beyond the graph (Na^+^ = 479/1955; Cl^−^ = 552/2084).

Our next question was whether the ionic pattern of the parasite reflected that of the host? This we checked by comparing the ion ratios in the parasite and the hosts. The increase of Na^+^ on halophytic hosts could also be deduced from the higher Na/K ratio on halophytic than non-halophytic hosts (see Table 2). Our samples also showed that the soluble fraction of Ca^2+^ (the hot water fraction, which is approximately the portion that is osmotically relevant) was very different between species (Table 3), where the two chenopod hosts caused much higher Ca^2+^ concentration in the parasite than in the other species (Table 3). The results indicate that the host's concentrations of osmotically active ions are not strongly correlated with the equivalent ions in the parasite (Table 4 and Fig. 12).

**Table 2.** Sodium–potassium ratio (derived from H_2_O values, mmol kg^−1^) in leaves of the non-halophytic hosts *A. tortilis, C. comosum* and *H. persicum* the halophytic hosts *T. nilotica, A. halimus* and *N. retusa* and the respective *P. acaciae* hemi-parasites on them.

Non-halophytic	Na/K	Halophytic	Na/K
*A. tortilis*		*T. nilotica*	
Leaves	0.37	Young shoots	0.65
*P. acaciae*		*P. acaciae*	
Old leaves	0.069	Old leaves	2.46
Middle-aged leaves	0.056	Middle-aged leaves	1.81
Young leaves	0.056	Young leaves	1.06
*C. comosum*		*A. halimus*	
Shoots	0.77	Leaves	5.90
*P. acaciae*		*P. acaciae*	
Old leaves	0.41	Old leaves	4.31
Middle-aged leaves	0.40	Middle-aged leaves	2.26
Young leaves	0.37	Young leaves	1.75
*H. persicum*		*N. retusa*	
Shoots	1.36	Leaves	3.30
*P. acaciae*		*P. acaciae*	
Old leaves	1.10	Old leaves	2.45
Middle-aged leaves	0.92	Middle-aged leaves	1.54
Young leaves	0.68	Young leaves	0.82

**Table 3.** Ion contents of old leaves of *P. acaciae* on various hosts (± standard deviation) and corresponding ionic contents of host plants [in each cell: upper figure, mmol kg^−1^ d.m.; lower figure, mmol kg^−1^ H_2_O].

Taxon: parasite and relevant host	Na^+^	K^+^	Ca^2+^	Mg^2+^	Cl^−^
*P. acaciae* (*N* =12)	45.9 ± 12.6	668 ± 201	307 ± 104	86.4 ± 19.7	220 ± 88.9
	26.6 ± 7.67	375 ± 63.9	181 ± 72.9	49.5 ± 8.01	126 ± 46.7
*A. raddiana* (*N* = 12)	55.7 ± 14.1	161 ± 89.4	519 ± 243	94.3 ± 24.9	82.3 ± 28.7
	44.7 ± 16.8	115 ± 35.7	440 ± 217	78.4 ± 31.3	63.6 ± 24.2
*P. acaciae* (*N* = 10)	38.0 ± 8.56	596 ± 296	454 ± 128	111 ± 32.2	248 ± 80.5
	23.2 ± 6.63	334 ± 125	274 ± 84.6	65.6 ± 15.2	144 ± 29.7
*A. tortilis* (*N* = 10)	47.6 ± 10.0	127 ± 53.3	607 ± 272	162 ± 51.5	82.5 ± 21.6
	40.3 ± 8.23	105 ± 37.2	528 ± 242	140 ± 49.8	70.7 ± 20.9
*P. acaciae* (*N* = 2)	35.9 ± 4.46	864 ± 199	283 ± 50.3	259 ± 46.1	140 ± 10.5
	14.4 ± 2.73	334 ± 27.5	111 ± 15.1	102 ± 13.9	56.9 ± 13.6
*A. lebbeck* (*N* = 2)	33.1 ± 1.06	269 ± 83.9	141 ± 77.9	149 ± 53.4	49.9 ± 13.0
	14.5 ± 2.52	112 ± 12.8	69.5 ± 47.2	70.8 ± 37.1	20.9 ± 1.24
*P. acaciae* (*N* = 10)	399 ± 176	997 ± 313	83.6 ± 60.4	312 ± 94.3	399 ± 190
	142 ± 59.2	350 ± 76.8	31.9 ± 25.3	112 ± 36.8	141 ± 65.8
*C. comosum* (*N* = 10)	108 ± 56.0	156 ± 98.4	51.9 ± 102	283 ± 92.0	79.0 ± 25.5
	70.1 ± 33.1	91.4 ± 44.3	60.0 ± 147	204 ± 109	52.9 ± 19.2
*P. acaciae* (*N* = 2)	822 ± 293	688 ± 139	41.0 ± 3.07	128 ± 21.1	789 ± 273
	247 ± 14.0	222 ± 48.9	13.9 ± 4.74	41.9 ± 10.7	238 ± 16.2
*C. cunninghamiana* (*N* = 2)	98.7 ± 15.5	255 ± 16.3	267 ± 2.04	195 ± 14.1	317 ± 106
	71.0 ± 21.6	181 ± 38.9	188 ± 27.3	135 ± 11.1	234 ± 108
*P. acaciae* (*N* = 7)	556 ± 126	496 ± 52.4	127 ± 38.9	265 ± 57.0	387 ± 173
	327 ± 59.6	295 ± 38.3	75.0 ± 23.6	160 ± 46.4	223 ± 86.0
*H. persicum* (*N* = 8)	941 ± 165	698 ± 113	7.23 ± 2.74	295 ± 39.4	479 ± 202
	572 ± 110	420 ± 43.8	4.44 ± 1.87	181 ± 37.5	290 ± 127
*P. acaciae* (*N* = 10)	329 ± 208	862 ± 184	111 ± 35.9	133 ± 28.6	166 ± 121
	145 ± 76.2	397 ± 61.4	52.6 ± 19.0	61.8 ± 13.7	73.4 ± 42.1
*O. baccatus* (*N* = 10)	129 ± 53.1	209 ± 60.5	117 ± 23.0	71.5 ± 14.8	85.4 ± 59.2
	109 ± 39.7	178 ± 36.6	102 ± 22.7	62.7 ± 15.7	69.6 ± 40.7
*P. acaciae* (*N* = 2)	74.1 ± 17.0	961 ± 253	249 ± 14.8	208 ± 33.0	745 ± 76.6
	25.6 ± 0.86	330 ± 0.69	90.3 ± 18.5	73.3 ± 7.84	267 ± 43.5
*Z. spina-christi * (*N* = 2)	67.2 ± 2.87	102 ± 52.9	258 ± 131	196 ± 32.3	385 ± 14.2
	36.1 ± 12.3	45.5 ± 11.7	116 ± 28.3	98.7 ± 14.3	207 ± 69.3
*P. acaciae* (*N* = 3)	2603 ± 231	609 ± 193	160 ± 74.0	271 ± 67.1	3006 ± 373
	479 ± 22.4	111 ± 30.1	30.1 ± 14.4	50.6 ± 14.1	552 ± 42.8
*A. halimus* (*N* = 3)	3267 ± 400	594 ± 171	11.2 ± 4.10	268 ± 46.0	3458 ± 430
	1955 ± 801	331 ± 86.1	6.28 ± 2.13	179 ± 122	2084 ± 902
*P. acaciae* (*N* = 10)	1481 ± 366	572 ± 182	344 ± 71.4	281 ± 42.6	1068 ± 302
	500 ± 77.4	204 ± 81.0	121 ± 36.0	97.0 ± 15.9	362 ± 81.3
*N. retusa* (*N* = 10)	1586 ± 762	457 ± 159	611 ± 155	513 ± 85.6	1560 ± 724
	446 ± 154	135 ± 48.9	179 ± 45.0	151 ± 27.0	442 ± 146
*P. acaciae* (*N* = 17)	1503 ± 531	621 ± 145	501 ± 105	632 ± 62.6	1382 ± 471
	300 ± 105	126 ± 35.1	102 ± 26.6	128 ± 18.1	273 ± 72.8
*T. aphylla* (*N* = 15)	586 ± 196	259 ± 58.6	504 ± 127	598 ± 166	573 ± 217
	306 ± 82.5	139 ± 37.8	274 ± 87.9	326 ± 107	299 ± 92.0
*P. acaciae* (*N* = 12)	2253 ± 466	917 ± 219	517 ± 67.9	291 ± 45.9	1621 ± 438
	402 ± 54.8	169 ± 52.2	94.2 ± 16.8	53.0 ± 10.7	289 ± 60.1
*T. nilotica* (*N* = 12)	240 ± 99.9	367 ± 67.1	590 ± 112	319 ± 52.1	413 ± 544
	169 ± 72.5	259 ± 46.5	417 ± 84.6	226 ± 42.0	287 ± 368

**Table 4.** Ion ratio (mmol-paras/mmol-host) calculated from H_2_O values (mmol kg^−1^) for the checked parasite/host pairs.

Ion ratio: parasite/host for taxon	Ratio for Na^+^	Ratio for K^+^	Ratio for Ca^2+^	Ratio for Mg^2+^	Ratio for Cl^−^
*A. raddiana* (*N* = 12)	0.60	3.26	0.41	0.63	1.98
*A. tortilis* (*N* = 10)	0.58	3.18	0.52	0.47	2.04
*A. lebbeck* (*N* = 2)	0.99	2.98	1.60	1.44	2.72
*C. comosum* (*N* = 10)	2.03	3.82	0.53	0.55	2.66
*C. cunninghamiana* (*N* = 2)	3.48	1.23	0.074	0.31	1.02
*H. persicum* (*N* = 8)	0.57	0.70	16.9	0.88	0.77
*O. baccatus* (*N* = 10)	1.33	2.23	0.52	0.99	1.05
*Z. spina-christi* (*N* = 2)	0.71	7.25	0.78	0.74	1.29
*A. halimus* (*N* = 3)	0.24	0.34	4.79	0.28	0.26
*N. retusa* (*N* = 10)	1.12	1.51	0.68	0.64	0.82
*T. aphylla* (*N* = 15)	0.98	0.91	2.91	0.39	0.91
*T. nilotica* (*N* = 12)	2.37	0.65	0.23	0.23	1.01

## Discussion

Previously published information on hosts of *P. acaciae* (Zohary 1966; Feinbrun-Dothan *et al.* 1991; Shmida and Darom 1992) mentioned *Atriplex* and *Tamarix* but no particular species. Qasem (2009, 2011) updated the list of hosts of *P. acaciae* in Jordan to 26 species from 12 families (included in Table 1). In total, there are now 37 non- and 6 halophytic hosts known. Similar to other halophytes, the succulence observed for *P. acaciae* growing on halophytic hosts showed a morphological adaptation of the mistletoe to salt stress (Breckle 1990). The increased succulence presumably resulted from absorption of Na^+^ and Cl^−^ ions from the host and osmotic adjustment by the parasite (Veste and Breckle 1995). There was a highly significant correlation between the succulence and Na^+^ and Cl^−^ accumulation, which is in accordance with observations made by Popp *et al.* (1995) for *T. oleifolius* growing on *Tamarix usnoides* in Namibia. An increase of Na^+^ can also be deduced from the higher Na/K ratio on halophytic than non-halophytic hosts (see Table 2), a very general rule for many other non-parasitic halophytes (Albert 1982; Reimann and Breckle 1993; Breckle 2002). Popp and Richter (1998) also reported an increase in Na/K ratios for *T. oleifolius* growing on *T. usneoides* from 1.58 to 13.21 with increasing leaf age.

The concentrations of osmotically active ions were not strongly correlated with those of the parasite. This may easily be explained by the fact that the samples of leaves from the parasite and of the corresponding young host stems or host leaves were not directly osmotically dependent. Similarly, the Na/K ratios did not reflect the relation between the host and the parasite (Tables 2 and 4).

It is difficult to be certain about the concentrations of ions available to the haustoria of the parasite, as obtaining xylem sap (the presumed source) is in itself difficult. However, the concentrations of monovalent ions in the leaves of the host are unlikely to provide a good indication of the xylem concentrations due to the presence of salt bladders (*Atriplex* species) or salt glands (*Tamarix* species). Additionally osmotic adjustment may involve compatible solutes (Yancey 1994; Flowers and Colmer 2008), which we did not investigate; they are, however, found in other mistletoes (Popp and Polania 1989), although in the more succulent leaves of *T. oleifolius* they are of minor importance for osmotic adjustment (Popp *et al.* 1995).

## Conclusions

Various definitions and classifications of halophytes have been proposed over the past decades, but following the classification of halophytes by Breckle (1990, 2000, 2002), which includes the mechanisms for controlling the NaCl concentration in plants, *P. acaciae* is a facultative eu-halophyte. *Plicosepalus acaciae* increases its halo-succulence according to the host, which is in this respect comparable with soil substrates. In any case, the water and nutrient status of the host plants, especially under desert conditions has a strong influence on the equilibrium of host and parasite and thus may mutually influence mortality (Bowie and Ward 2004). Surface structure, size and indirectly host origin influence the success of germination and of seedling establishment of parasite on host twigs (Rödl and Ward 2002): whether, during these early stages of development, the halophytic character of the host plant and salinity plays a role is still unclear. Host specificity seems to be rather low in mistletoes (Norton and Carpenter 1998; Norton and de Lange 1999) since seeds germinate readily in almost all situations, whereas other parasitic plants germinate only in response to chemical signals from the host plants. In future a more detailed analysis of xylem concentrations and ion patterns of host and parasite is a strong necessity for a better understanding of ecophysiological behaviour and the differing infestation rates of the parasite on different species of the host. But our results show that the ion pattern of substrate (host) and plant (parasite) is, to some extent, mirrored, but that haustoria of the parasite (similar to roots in soil) have only a limited capacity for ion selectivity. Thus, we can conclude that the mistletoe we studied exhibited an adaptive plasticity depending on substrate conditions offered by the host, reflecting the xylem sap concentrations of Na^+^ and Cl^−^. Basically this host–parasite association is an excellent model system and could be used for future ecophysiological research on halophytes.

## Sources of Funding

The research was funded by DAAD—Deutscher Akademischer Austauschdienst, Bonn, Germany, and the BMBF—Federal Ministry of Science and Education, Bonn, Germany.

## Contributions by the Authors

All authors have contributed substantially to this manuscript. M.V. was involved in planning, data analyses and manuscript writing. H.T. conducted the field investigations, and was involved in the data analysis and manuscript writing. S-W.B. was involved in the planning and supervision of all the experimental work and in writing the manuscript.

## Conflicts of Interest Statement

None declared.
